# Profiling prefrontal cortex protein expression in rats exhibiting an incubation of cocaine craving following short-access self-administration procedures

**DOI:** 10.3389/fpsyt.2022.1031585

**Published:** 2023-01-04

**Authors:** Laura L. Huerta Sanchez, Mathangi Sankaran, Taylor L. Li, Hoa Doan, Alvin Chiu, Eleanora Shulman, Gabriella Shab, Tod E. Kippin, Karen K. Szumlinski

**Affiliations:** ^1^Department of Psychological and Brain Sciences, University of California, Santa Barbara, Santa Barbara, CA, United States; ^2^Department of Molecular, Cellular and Developmental Biology, University of California, Santa Barbara, Santa Barbara, CA, United States; ^3^Neuroscience Research Institute, University of California, Santa Barbara, Santa Barbara, CA, United States

**Keywords:** incubation of craving, prelimbic cortex, infralimbic cortex, Akt, glutamate receptors, Homer proteins

## Abstract

**Introduction:**

Incubation of drug-craving refers to a time-dependent increase in drug cue-elicited craving that occurs during protracted withdrawal. Historically, rat models of incubated cocaine craving employed extended-access (typically 6 h/day) intravenous drug self-administration (IV-SA) procedures, although incubated cocaine craving is reported to occur following shorter-access IV-SA paradigms. The notoriously low-throughput of extended-access IV-SA prompted us to determine whether two different short-access IV-SA procedures akin to those in the literature result in qualitatively similar changes in glutamate receptor expression and the activation of downstream signaling molecules within prefrontal cortex (PFC) subregions as those reported previously by our group under 6h-access conditions.

**Methods:**

For this, adult, male Sprague-Dawley rats were trained to intravenously self-administer cocaine for 2 h/day for 10 consecutive days (2-h model) or for 6 h on day 1 and 2 h/day for the remaining 9 days of training (Mixed model). A sham control group was also included that did not self-administer cocaine.

**Results:**

On withdrawal day 3 or 30, rats were subjected to a 2-h test of cue-reinforced responding in the absence of cocaine and a time-dependent increase in drug-seeking was observed under both IV-SA procedures. Immunoblotting of brain tissue collected immediately following the cue test session indicated elevated phospho-Akt1, phospho-CaMKII and Homer2a/b expression within the prelimbic subregion of the PFC of cocaine-incubated rats. However, we failed to detect incubation-related changes in Group 1 metabotropic glutamate receptor or ionotropic glutamate receptor subunit expression in either subregion.

**Discussion:**

These results highlight further a role for Akt1-related signaling within the prelimbic cortex in driving incubated cocaine craving, and provide novel evidence supporting a potential role also for CaMKII-dependent signaling through glutamate receptors in this behavioral phenomenon.

## Introduction

According to the most recent World Drug Report (1), ~ 20 million people worldwide, aged 15–64 years, have used cocaine in the past year, and there has been a 30–50% rise in the global manufacturing, trafficking and seizure of cocaine over the past 10 years. In certain individuals, cocaine use can result in cocaine use disorder -a chronic, relapsing, brain disease characterized by cycles of binging, withdrawal, craving and resumption of drug-taking (2). The presentation of drug-associated stimuli is well-characterized to elicit a subjective desire for drug (i.e., drug craving) that can lead to relapse (3, 4) and the intensity of cue-elicited craving intensifies insidiously during the course of drug abstinence (5–12). This “incubation of craving” [(13); here on in referred to simply as “incubated craving”] is posited to underpin the chronic, relapsing, nature of substance use disorder by driving perseverative cue hyper-reactivity even during protracted drug withdrawal or abstinence (14–16).

Multi-modal pharmacotherapeutic strategies exist to mitigate drug-craving and prevent relapse in those suffering from opioid, nicotine and alcohol use disorders. No FDA-approved pharmacotherapeutic exists to curb cocaine craving and treat cocaine use disorder. As such, we examine the neurobiological correlates of incubated craving in an effort to elucidate potential druggable targets for prolonged management of cocaine use disorder using an animal model.

Studies of incubated cocaine craving using rat models are in growing use and generally involve access to cocaine followed by testing at various periods without cocaine access. Typically, rats undergo daily intravenous cocaine self-administration (IV-SA) sessions for a proscribed period, during which each cocaine delivery is associated with a compound light-tone stimulus. Then, during either early or later withdrawal (e.g., 1–3 days) or later withdrawal (e.g., 2 weeks up to 6 months), the secondary reinforcing properties of the cocaine-associated cues are evaluated in the absence of cocaine. In particular, use of “extended-access” (i.e., 6 h/day) self-administration regimens to proceed tests of incubated cocaine craving were initially employed by Grimm et al. over 20 years ago and has proven to have high reliability and reproducibility both within and across many laboratories [e.g., (17–26)], including our own (27–33). Moreover, there is congruency in findings from different laboratories with respect to anomalies in corticoaccumbens glutamate signaling as being central to incubated behavior, but not drug-craving *per se* [e.g., (21, 22, 30, 31, 34)]. Consequently, the majority of our knowledge of incubation and its underlying biology is limited to extended-access (i.e., 6 h/day) IV-SA training conditions.

However, the time-dependent intensification of cue-elicited cocaine craving appears to generalize across self-administration conditions. In fact, time-dependent increases in cocaine seeking were first reported in rats trained under a 3-h/day cocaine IV-SA paradigm (35). Similarly, others have reported incubated cocaine craving following daily 2- or 12-h access procedures (36). Further, the first ever study of sex/reproductive cycle differences in incubated cocaine seeking demonstrated that a daily 2-h IV-SA procedure is sufficient to elicit incubated cocaine craving in both male and female rats (37), the findings of which were extended to daily extended-access (6 or 8-h) IV-SA or daily intermittent IV-SA procedures (18, 38). Consistent with these earlier indications that the manifestation of incubated cocaine craving is reliably observed across different durations of daily access to self-administration (i.e., not specifically tied to 6+ h/day) IV-SA procedures, other studies have reported an incubation of cocaine craving following a “mixed” IV-SA model in which rats were allowed over-night access to cocaine on the first day of self-administration training, but then underwent 1 or 2-h IV-SA sessions for the remainder of the self-administration phase of the study [e.g., (39–43)]. Further, under such mixed IV-SA procedures, incubated cocaine craving is associated with time-dependent anomalies in the activational state of corticoaccumbens projections—findings that align with glutamate-associated perturbations within the nucleus accumbens core (NAC core) and vmPFC reported under the 6-h IV-SA model [e.g., (17, 21, 22, 25, 27, 30, 31, 44–46)].

Indeed, our group recently conducted a series of experiments in male rats employing a more procedurally facile mixed IV-SA model in which rats were allowed 6-h access to cocaine on day 1 of self-administration training, followed by 9 days of 2-h access to the drug in order to increase the throughput of our studies (47). This procedure (here on in referred to as Mixed or mixed-access) was not only sufficient to induce incubated cocaine craving but also to elicit incubation-selective changes in the protein expression within the prelimbic (PL) subregion of the PFC (47) akin to those reported by our group to occur within the vmPFC of rats with a history of 6-h IV-SA procedures (27, 28, 33)—notably an increase in Homer2a/b expression and an increased indices of mTOR activation. Such results lead to the hypothesis that common molecular adaptations, particularly within the PL, might underpin the incubation of cocaine-craving, irrespective of the self-administration procedure employed to elicit said craving. However, the rats in our prior report (47) were gavage-infused with the mTOR inhibitor Everolimus or its vehicle prior to testing and tissue collection. Thus, we sought to replicate/extend our findings of time-dependent changes at the behavioral and neurobiological levels to the Mixed model (in the absence of any other additional experimental manipulations), as well as a limited-access (2 h/day) model reported previously to result in incubated cocaine-craving (37).

Specifically, we examined our two models for incubation-related increases in both Akt1 phosphorylation (an index of PI3K/mTOR activity; 33) and the expression of the glutamate receptor scaffolding protein Homer2a/b (28, 47), as well as reduced expression of the Group1 metabotropic glutamate receptors mGlu1 and mGlu5 (27). While we have previously demonstrated that extended-access cocaine IV-SA elevates the expression of GluN2a and GluN2b within the mPFC of rats at 60 days withdrawal (48), to the best of our knowledge, no study has examined for changes in AMPA or NMDA receptor subunits within PFC subregions of rats expressing incubated cocaine seeking. Thus, we also examined for time-dependent changes in the expression of the obligatory subunits of both receptors (GluA1 and GluN1), as well as subunits known to regulate channel properties (e.g., GluA2, GluN2a, GluN2b) [c.f., (49, 50)]. Finally, we also examined for changes in CaMKII phosphorylation as an index of calcium-dependent signaling (51).

## Methods

### Subjects

Adult male Sprague-Dawley rats (250–275 g upon arrival) were obtained from Charles River Laboratories (Hollister, CA, USA). Rats were housed in a colony room, controlled for temperature and humidity, under a 12-h day/ 12-h night cycle (lights off from 07:00 to 19:00) hours. Rats were given *ad libitum* access to food and water throughout the duration of the study and were allowed to acclimate to the colony room for 48 h following arrival prior to handling. All experimental protocols were consistent with the guidelines of the *NIH Guide for Care and Use of Laboratory Animals* (NIH publication No. 80-23, revised 2014) and were reviewed and approved by the University of California, Santa Barbara Institutional Animal Care and Use Committee under protocol 829.2. A total of 22 rats were excluded from the statistical analyses of the data for failing to meet the IV-SA acquisition criterion detailed below.

### Surgical procedures

Rats slated to undergo cocaine IV-SA underwent a surgery to implant chronic IV catheters as previously described [e.g., (47)]. Rats were placed under ketamine/xylazine anesthesia (11.76 mg/ml xylazine and 88.23 mg/ml ketamine, Abbot Laboratories, North Chicago, IL, USA) and then each implanted with a chronic silastic catheter (13 cm long; 0.3 mm inner diameter, 0.64 mm outer diameter) into the right jugular vein. Banamine (2 mg/kg) and buprenorphine (0.3 mg/ml) were administrated subcutaneously to treat post-surgical pain. Bupivicaine (5 mg/ml) was administrated subcutaneously at the site of each incision prior to the start of the surgery, to act as a local analgesic. The catheter ran subcutaneously around the shoulder to the back where it lay perpendicular to the dorsal surface and was secured to a threaded 22-gauge metal guide cannula (Plastics One, Roanoke, VA, USA). A guide cannula protruded through a small hole on the animal's back and was capped (when not in use) to protect against infection. The cannula was held in place by being cemented to a small (~0.5 × 0.5 inch) swatch of bard mesh (C. R. Bard Inc., Cranston, RI, USA). After the IV catheterization procedure, the catheters were immediately flushed with 0.1 ml of sterile cefazolin/ heparin (100 mg/ml cefazolin and 70 U/ml heparin) and 0.1 ml of sterile gentamicin (2 mg/ml). During the first 2-days of post-operative care, animals received sub-cutaneous banamine twice a day. For the remainder of the study, animals received daily IV injections of gentamicin and cefazoin/heparin to maintain catheter patency. Prior to the beginning of self-administration training, catheter patency was verified through IV administration 0.1 ml of sodium Brevital (10 mg/ml), which produces rapid loss of muscle tone when administered intravenously. Catheter patency was verified again at the end of self-administration prior to conducting tests for cue-reinforced behavior. To reduce the potential for subject attrition due to catheter failure, control rats underwent a sham surgical procedure involving the administration of the same anesthesia and analgesics as cocaine IV-SA rats, but only received one dorsal and one ventral incision, which were then immediately stapled. All animals were given a minimum of 4 days for recovery prior to the start of self-administration.

### 2-h IV-SA procedure

The 2-h IV-SA procedure (2-h) was based on procedures described in Kerstetter et al. (37) in which both male and female rats exhibited incubated responding, with the exception that no prior food-reinforced lever-response training was employed in our study to facilitate data interpretation. Under our 2-h procedure, rats were trained to lever-press under a fixed interval 20 (FI20) schedule of reinforcement for a 5-s IV infusion of a 0.25 mg/kg/0.1 ml cocaine solution during daily 2-h sessions over 10 days. Depression of the cocaine-reinforced, active, lever also activated a 20-s light and tone (78 dB, 2 kHz) compound stimulus, which also served as a time-out period in which lever presses were recorded but had no consequences. Depression of the non-reinforced, inactive, lever had no programmed consequences. To prevent over-dose, the number of cocaine infusions during the first day was capped at 100 infusions (48). Sham controls were simply placed into the operant chambers for 2 h/day and their responding on the active lever resulted in the presentation of the light/tone compound stimulus only. Following self-administration, rats were assigned to be tested on either withdrawal day 3 or 30 (respectively, WD3 or WD30) in such a way as to ensure that cocaine intake was comparable between the two test days. Cocaine-reinforced rats that failed to respond for at least 15 infusions and failed to exhibit 75% of their total responding on the active vs. inactive lever over the last 3 days of self-administration were excluded from the study and did not undergo cue-reinforced testing. Rats that successfully acquired cocaine self-administration remained in their home cages for their assigned period of abstinence/withdrawal, after which tests for cue-reinforced responding were conducted (see below). There were no criteria for Sham rats to advance to testing and thus, all Sham rats were tested for incubated craving.

### Mixed IV-SA procedure

The other IV-SA procedure (**Mixed**) tested in this study was identical to that described in Chiu et al. (47). Following IV catheterization, rats underwent a 6-h self-administration session on Day 1 of training and for the next 9 days, rats underwent the same 2-h self-administration training procedures as those described for the 2-h model (see Section **2**-h IV-SA procedure) and were randomly assigned to either the WD3 or WD30 test groups in such a way as to ensure comparable cocaine intake over the last 3 days of self-administration. As for the 2-h model, cocaine-reinforced rats that failed to respond for at least 15 infusions and failed to exhibit 75% of their total responding on the active vs. inactive lever over the last 3 days of self-administration were excluded from the study and did not undergo cue-reinforced testing and, following the tenth day of self-administration, rats were returned to their home cages for their designated period of abstinence/withdrawal at which time, tests for cue-reinforced responding was conducted as described below.

### Tests for incubated craving

**Figure 1:**
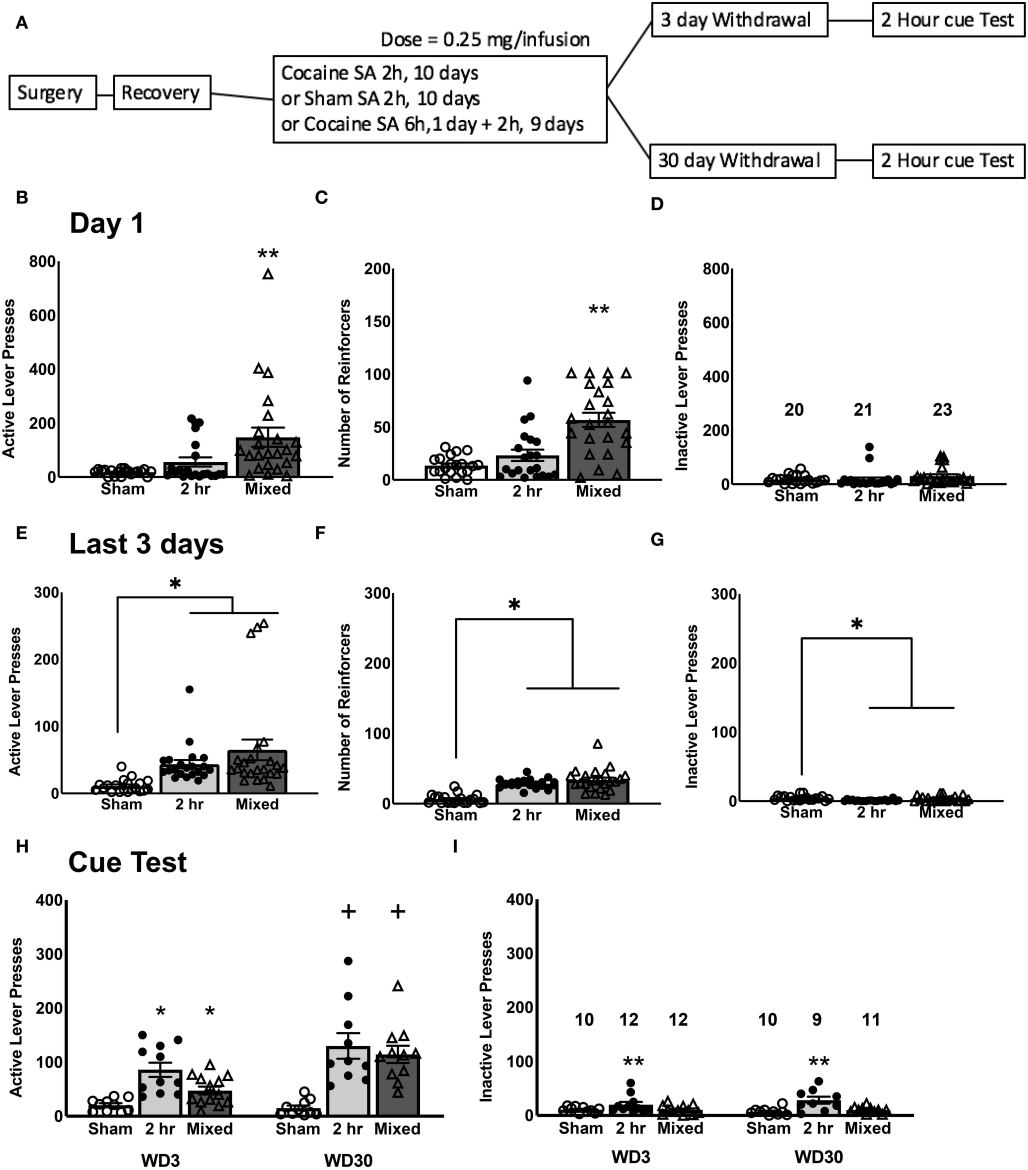
Cocaine self-administration and test for incubated craving. **(A)** Experimental timeline of the study. On day 1 of self-administration training, Mixed rats that underwent a 6-h cocaine IV-SA session exhibited more active lever-presses **(B)**, received more reinforcers **(C)**, and exhibited more inactive lever-presses **(D)**, compared to both Sham controls and 2-h rats that underwent a 2-h cocaine IV-SA session. By the last 3 days of self-administration training, rats in both IV-SA groups exhibited a comparable number of active lever-presses **(E)**, reinforcers earned **(F)**, and inactive lever-presses **(G)**, which were higher than Sham controls. **(H)** On the test for incubated cocaine craving, both cocaine IV-SA groups showed a time-dependent increase in cue-elicited active lever-pressing from WD3 to WD30. **(I)** There were no time-dependent changes in inactive lever-pressing for any groups. Data is represented as means ± SEMs of the number of rats indicated. *p < 0.05, compared to Sham, **p < 0.05, compared to other two groups, +*p* < 0.05, compared to WD3 (incubation).

On either WD3 or WD30, rats underwent a 2-h test for incubated cocaine craving under extinction conditions. The final sample sizes of each group tested for incubated cocaine craving were as follows: for WD3: *n* = 10 Sham, *n* = 12 2-h, and *n* = 12 Mixed; for WD30: *n* = 10 Sham, *n* = 9 2-h, and *n* = 11 Mixed. During the test for cue-elicited craving, depression of the active lever resulted in the presentation of the 20-s light/tone cue but no cocaine delivery (i.e., was conducted under extinction conditions). Incubated cocaine craving was operationally defined as a significant increase in the number of cue-reinforced active lever responses on WD30 vs. WD3. Immediately following cue-testing, rats were decapitated, the prelimbic (PL) and infralimbic (IL) cortices were dissected over ice (47) and stored frozen at −80°C until immunoblotting. A schematic of the procedural time-line employed in this study is provided in Figure 1A.

### Immunoblotting

To determine whether or not the protein correlates of incubated craving induced by our shorter access IV-SA procedures were qualitatively similar to those induced under 6-h IV-SA procedures (27, 28, 33) or under the mixed IV-SA model (47), immunoblotting was conducted on the PL and IL of our Sham, 2-h and Mixed-access rats tested for cue-elicited cocaine craving on WD3 and WD30. The procedures for preparing tissue homogenates, detecting and quantifying the expression of our proteins of interest were similar to those employed in our recent study (47). Herein, the following rabbit primary antibodies were used: Homer2a/b (1:1,000 dilution; Synaptic Systems; 160 203), mGlu5 (1:1,000 dilution; Millipore; AB5675), Akt1 (1:1,000 dilution; Cell Signaling Technology; 9272), GluN1 (1:250 dilution; Cell Signaling Technology; 5704S), GluN2a (1:250 dilution; Millipore; 07-632) and GluA1 (1:200; Millipore; AB1504) and p(Thr286)-CaMKII (1:500 dilution; Cell Signaling Technology; 3361). The following mouse primary antibodies were also employed: Homer1b/c (1:1,000 dilution; Santa Cruz Biotechnology; sc-25271), mGlu1 (1:1,000 dilution; BD Biosciences; 610965), p(Ser473)-Akt1 (1:1,000 dilution; Cell Signaling Technology; 4051), GluN2b (1:500 dilution; Invitrogen; MA1-2014), GluA2 (1:500 dilution; Synaptic Systems; 182 111), and CaMKII (1:500 dilution; Millipore; 05-532). As conducted in prior our work, calnexin expression was employed to control for protein loading and transfer using a rabbit primary anti-calnexin antibody (1:1,000 dilution; Enzo Life Sciences; ADI-SPA-860). Membranes were subsequently washed with PBST, incubated in either a goat anti-rabbit IRDye 800 CW secondary antibody (1:10,000 dilution; Li-Cor; 925-3221) or a goat anti-mouse IRDye 680RD secondary antibody (1:10,000 dilution; Li-Cor; 925-68070), dried, and imaged on an Odyssey Infrared Imaging System (Li-Cor Biosciences, Lincoln, NE, USA). Raw values for each band were measured, and first normalized to their corresponding calnexin signal and then to the average value of the Sham control (i.e., Sham-3WD). As our selected mGlu1 antibody failed to reliably detect the dimer form of the receptor (see Supplementary Figure 1), only the monomer form of this receptor is reported herein. All immunoblots were visually inspected prior to image analyses for signal anomalies including: lack of signal (i.e., blank blot/lane with corresponding missing calnexin signal), misshaped band (e.g., abnormal bright spot vs. rectangular or bowtie-shaped band), high or splotchy background around blot and indices of air bubbles from transfer (i.e., black spots in the blot). Blots exhibiting any of these anomalies were not included in the final statistical analyses of the data.

### Statistical analyses

For the behavioral aspects of this study, responding on the active and inactive lever, as well as the number of cocaine reinforcers earned served as the dependent variables of interest. For each dependent variable, a univariate two-tailed Group (Sham, 2-h, Mixed) X Withdrawal (WD3 vs. WD30) analysis of variance (ANOVA) was conducted on Day 1 of self-administration training to compare behavior between rats allowed 2- vs. 6-h access to cocaine and a similar analysis was conducted on the average of the last 3 days of self-administration training to ensure equivalent responding prior to testing. To test for withdrawal-dependent changes in behavior on the cue tests, orthogonal contrasts (52) were conducted between the rats tested on WD3 vs. WD30, separately for Sham, 2-h and Mixed rats and Cohen's *d* estimations of effect size were also determined (53). This latter analysis was one-tailed as it was expected that both IV-SA procedures would elicit an incubation of responding, based on prior work from our group (37, 47). For immunoblotting, the data were expressed as a percent of the Sham-WD3 control and then analyzed using two-tailed univariate Group X Withdrawal ANOVAs. Significant interactions were deconstructed along the Group factor and the means for WD3 and WD30 were compared using tests for simple main effects to determine which self-administration group exhibited a time-dependent change in protein expression during withdrawal (52). Pearson's correlational analyses were also conducted to determine the relationship between drug-seeking behavior and protein expression within PFC subregions as conducted previously (29, 47). For all analyses, significant interactions were followed up with tests for simple effects, and Tukey's *post-hoc* tests were applied following main effects, when appropriate.

## Results

### Self-administration training

As expected, given the difference in the duration of the first self-administration session, a comparison of the number of active lever-presses (Figure 1B), as well as reinforcers earned (Figure 1C), on Day 1 of self-administration was highest in the Mixed group. This was confirmed by significant Group effect for both variables [for active lever, *F*_(2, 63)_ = 7.685, *p* = 0.001; for reinforcers, *F*_(2, 63)_ = 19.010, *p* < 0.0001], coupled with the results of *post-hoc* tests (for both variables, Tukey's test: Mixed vs. 2-H, *p*'s < 0.0001; Mixed vs. Sham, *p*'s < 0.0001; 2-H vs. Sham, *p*'s > 0.10). While the Mixed rats emitted approximately double the number of inactive lever-presses on Day 1 as compared to the 2-H and Sham rats (Figure 1D), this difference was not statistically significant [*F*_(2, 63)_ = 1.326, *p* = 0.273]. As expected, we detected no Withdrawal effect or interactions for Day 1 behavior for the number of active or inactive lever-presses or for the number of reinforcers earned (for all variables, Withdrawal effect and interactions, all *p*'s > 0.250). As such, the data were collapsed across the two withdrawal periods for clarity of presentation of group difference in Day 1 responding (Figures 1B–D). However, by the end of the 10-day self-administration training phase of the study, both cocaine groups exhibited a similar level of behavior, which was different from that exhibited by the Sham controls (Figures 1E–G) [for active lever, *F*_(2, 63)_ = 6.912, *p* = 0.002; for reinforcers, *F*_(2, 63)_ = 38.114, *p* < 0.0001; for inactive lever, *F*_(2, 63)_ = 6.234, *p* = 0.003; for all 3 variables, Tukey's test: Mixed vs. 2-H, *p*'s = 0.250; Mixed vs Sham, *p* = 0.004; 2-H vs. Sham, *p* = 0.012]. Again, we detected no Withdrawal effect or interactions for the average behavior over the last 3 days of self-administration training (for all variables, Withdrawal effect and interactions, all *p*'s > 0.400) and thus, the data were collapsed across the two withdrawal periods to facilitate visualization of group differences prior to cue-testing (Figures 1E–G). From these data, we conclude that a single 6-h cocaine IV-SA session does not substantially alter subsequent cocaine reinforcement expressed under 2-h access conditions, relative that exhibited following training exclusively under 2-h access conditions.

### Test for incubated craving

Both cocaine IV-SA groups exhibited a time-dependent increase in active lever-pressing from WD3 to WD30, which was not apparent in the Sham controls (Figure 1H). This was supported by the results of orthogonal contrasts, conducted separately for each group [for Sham, *t*_(18)_ = 0.910, *p* = 0.187; for 2-h, *t*_(19)_ = 2.040, *p* = 0.027; for Mixed, *t*_(21)_ = 3.746, *p* = 0.0005]. However, it should be noted that a calculation of Cohen's *d* for each of the two IV-SA groups indicated relatively large effect sizes and the effect size for the Mixed group (*d* = 1.542) was larger than that for the 2-h group (*d* = 0.862) and in inspection of Figure 1H suggested that the difference in incubation magnitude between the two cocaine IV-SA groups reflected the higher responding of the 2-h rats on WD3, as responding on WD30 was comparable between Mixed and 2-h rats. Indeed, a closer examination of the WD3 data for the 2 h rats revealed that 4 out of 7 rats from the first cohort of 2-h rats responded 100+ times on the active, cue-reinforced, lever on WD3. In contrast, only 1 out of the 5 rats from the second cohort exhibited such a high level of responding at this time-point and a similar inter-cohort effect was apparent also for inactive lever-responding. To determine whether or not inter-cohort variability significantly contributed to our behavioral results during the tests for incubated craving, the contrasts were re-evaluated, adjusting for cohort as a covariate. None of these analyses indicated any significant cohort effect [for active lever-presses, Replicate effect: for Sham, *p* = 0.165; for 2-h, *p* = 0.473; for Mixed, *p* = 0.492; for inactive lever-presses, Replicate effect: for Sham, *p* = 0.937; for 2-h, *p* = 0.136; for Mixed, *p* = 0.890). In contrast, no time-dependent change in inactive lever-pressing was detected in any group (Figure 1I; *t*-tests: for Sham, *p* = 0.187; for 2-h, *p* = 0.084; for Mixed, *p* = 0.457). These data indicate that both short-access IV-SA procedures induce a time-dependent increase in responding that is selective for the cocaine cue-reinforced lever.

### Immunoblotting in the prelimbic cortex (PL)

#### Group 1 mGluRs and Homer proteins

**Figure 2:**
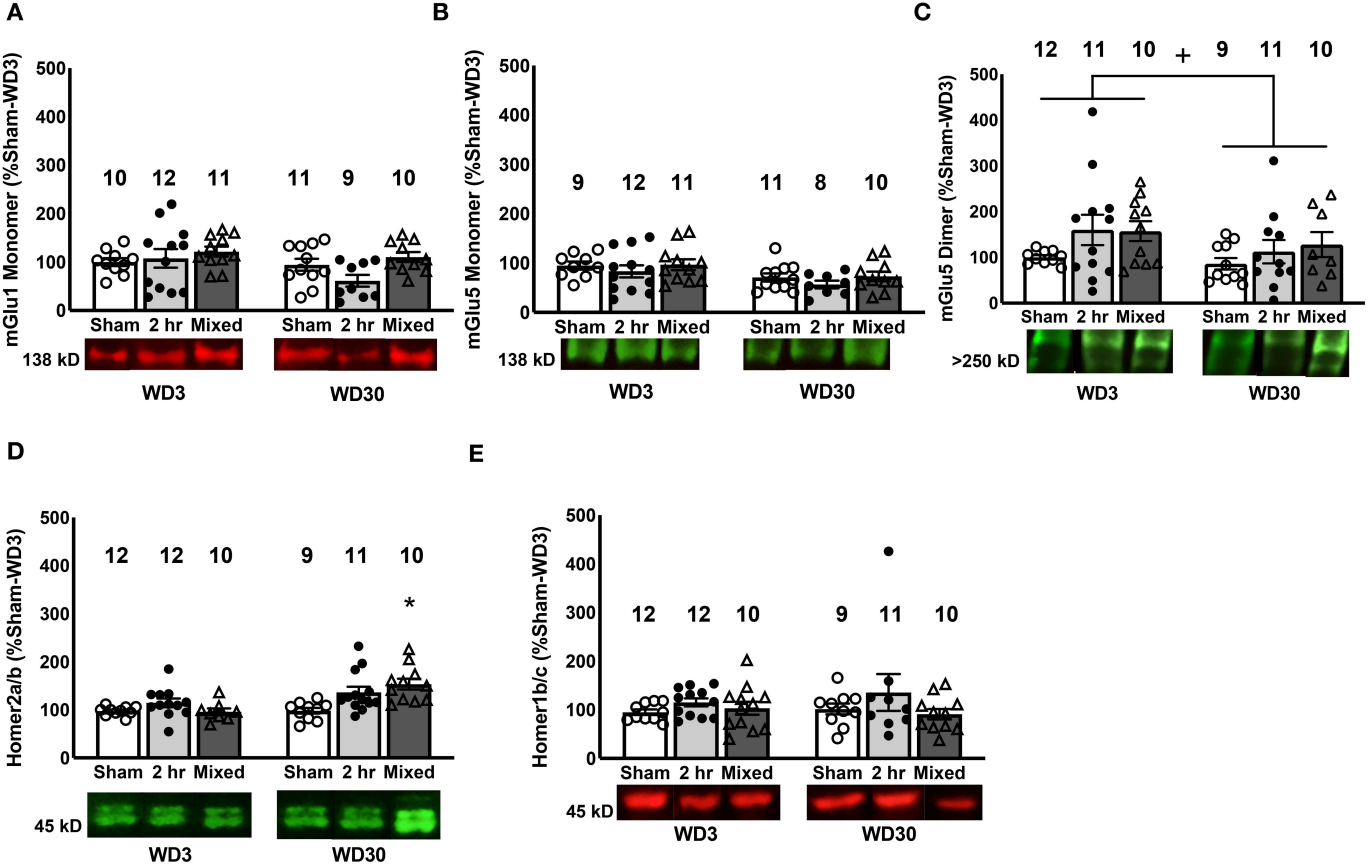
Changes in mGlu1/5 and Homer proteins in the PL. We detected no changes in the expression of mGlu1 **(A)** or the mGlu5 monomer **(B)** in the PL. **(C)** Irrespective of cocaine history, rats tested on WD30 exhibited lower PL expression of the mGlu5 dimer, compared to those tested on WD3. **(D)** Relative to Sham controls, only rats that underwent the Mixed IV-SA procedure exhibited a significant time-dependent increase in Homer2a/b within the PL. **(E)** No group differences in Homer1b/c expression were observed within the PL. Data are normalized to the average protein densities of the Sham controls tested on WD3 and represented as means ± SEMs of the number of rats indicated. *p < 0.05, compared to Sham, +*p* < 0.05, compared to WD3.

In contrast to our prior study employing the 6-h model (27), but consistent with our recent report using the mixed-access model (47), we detected no significant group differences in the PL expression of the mGlu1 monomer (Group effect and interaction, p's>0.060), although mGlu1 monomer expression tended to be lower overall on WD30 vs. WD3 (Figure 2A; Withdrawal effect: *p* = 0.055). Also consistent with Chiu et al. (47), no cocaine- or time-related differences in the expression of the monomer form of mGlu5 were detected within the PL (Figure 2B; Group X Withdrawal ANOVA: all *p*'s > 0.120), although mGlu5 dimer expression was significantly lower overall in the rats tested on WD30 vs. WD3 (Figure 2C) [Withdrawal effect: *F*_(1, 62)_ = 7.922, *p* = 0.007; Group effect and interaction, *p*'s > 0.258]. However, in contrast to both Miller et al. (29) and Chiu et al. (47), no significant correlations were noted between cue-elicited responding and the PL levels of either mGlu receptor monomer (for mGlu1 monomer, *r* = −0.005, *p* = 0.968; for mGlu5 monomer, *r* = −0.098, *p* = 0.454) or the mGlu5 dimer (*r* = −0.203, *p* = 0.111). Taken together with the results of Chiu et al. (47), the incubation of cocaine craving under short-access IV-SA procedures is insufficient to elicit changes in the PL expression of Group 1 mGlu receptors.

While the present findings with respect to the PL expression of Group1 mGluRs did not recapitulate those observed previously (27, 29, 47), a withdrawal-dependent increase in PL Homer2a/b expression was detected in both the 2-h and Mixed models (Figure 2D), in a manner akin to that reported for the entire vmPFC under the 6-h model (28) and the PL under the mixed model (47) [Group X Withdrawal: *F*_(2, 63)_ = 4.19, *p* = 0.02]. However, follow-up analyses revealed that this incubation-related increase in PL Homer2a/b expression was statistically significant only for the rats trained under the Mixed model (Figure 2D) [tests for simple effects: for Mixed, *p* < 0.05; for 2-h, *p* > 0.05; for Sham, *p* > 0.05]. Also consistent with our prior report (47), PL Homer2a/b expression was positively correlated with cue-elicited responding on test day [*r* = 0.323, *p* = 0.009]. In contrast, no change in PL Homer1b/c levels were detected (Figure 2E; Group X Withdrawal ANOVA, all *p*'s > 0.150) and no correlation was observed between PL Homer1b/c expression and cue-elicited responding on the test days [*r* = 0.021, *p* = 0.87]. These findings indicate that PL Homer2a/b expression is a common biomolecular correlate of cocaine craving across IV-SA models, and provide further evidence that incubation induced under mixed-access IV-SA procedures are sufficient to augment PL Homer2a/b expression.

#### iGluR subunits

**Figure 3:**
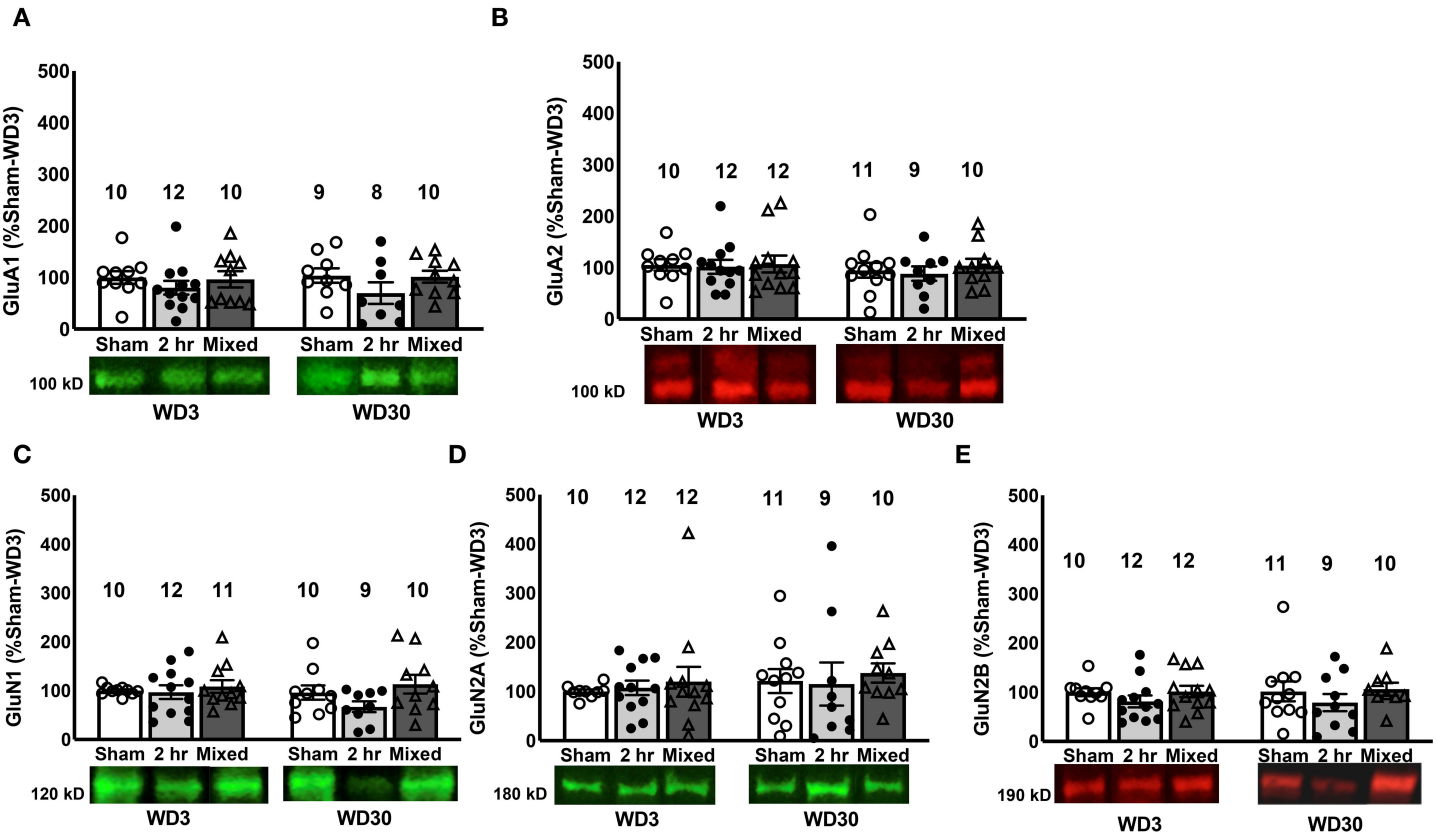
Changes in iGluR subunits in the PL. There were no differences between Sham, 2-h and Mixed rats with respect to the expression of the AMPA receptor subunits GluA1 **(A)** and GluA2 **(B)** within the PL, nor did we detect group differences in the NMDA subunits, GluN1 **(C)**, GluN2a, **(D)** or GluN2b **(E)**. Data are normalized to the average protein densities of the Sham controls tested on WD3 and represented as means ± SEMs of the number of rats indicated.

Immunoblotting for GluA1 and GluA2 within the PL failed to detect group differences in either AMPA receptor subunit (Figures 3A, B; Group X Withdrawal ANOVAs, for GluA1: all *p*'s > 0.062; for GluA2, all *p*'s > 0.233) and we detected no significant correlations between cue-elicited responding and their expression within the PL (for GluA1, *r* = −0.129, *p* = 0.367; for GluA2, *r* = 0.072, *p* = 0.571). Similarly, we detected no group differences in GluN1, GluN2a or GluN2b subunit expression within the PL (Figures 3C–E; Group X Withdrawal ANOVAs: for GluN1, all *p*'s > 0.109; for GluN2a, all *p*'s > 0.120; for GluN2b, all *p*'s > 0.078), with no correlations apparent between PL NMDA receptor subunit expression and behavior (for GluN1, *r* = 0.048, *p* = 0.713; for GluN2a, *r* = 0.020, *p* = 0.875; for GluN2b, *r* = −0.016, *p* = 0.902). These data argue that, in contrast to the NAC core [e.g., (17, 19, 54)], incubated cocaine craving is unrelated to changes in the total protein expression of these AMPA or NMDA subunits within the PL.

#### Akt and CaMKII

**Figure 4:**
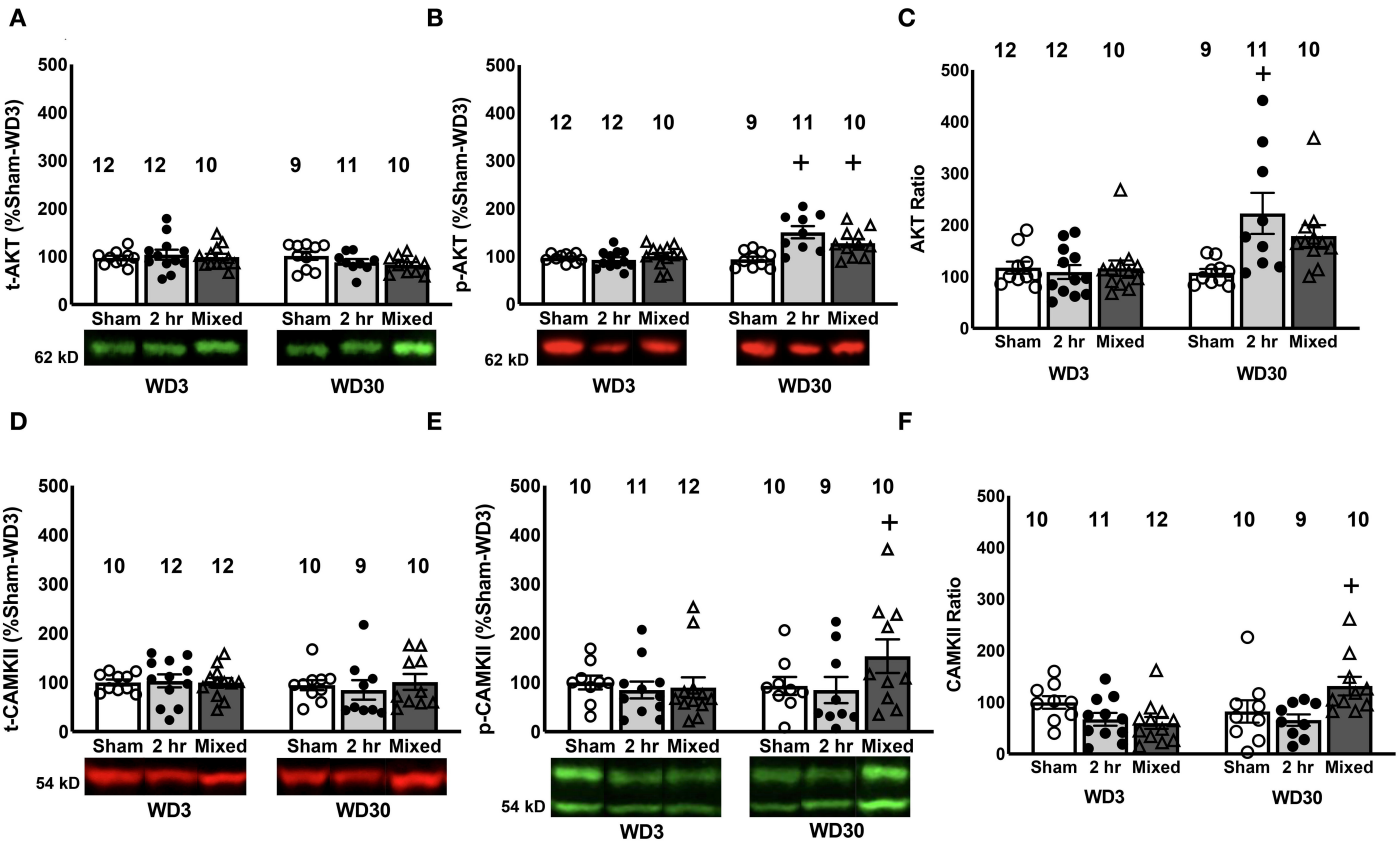
Changes in Akt1 and CaMKII expression and phosphorylation in the PL. While we did not detect any time-dependent or group differences in the PL expression of total Akt1 **(A)**, both 2-h and Mixed rats exhibited a time-dependent increase total p(Ser473)-Akt1 expression **(B)** and the 2-h rats exhibited a time-dependent increase in the relative expression of p(Ser473)-Akt1 **(C)**. **(D)** We detected no group or time-dependent changes in total CaMKII expression within the PL. However, the Mixed group demonstrated a significant time-dependent increase in both total **(E)** and relative **(F)** p(Thr286)-CaMKII expression. Data are normalized to the average protein densities of the Sham controls tested on WD3 and represented as means ± SEMs of the number of rats indicated. +*p* < 0.05, compared to WD3.

Total AKT levels were unchanged in the PL (Figure 4A; Group X Withdrawal ANOVA, all *p*'s > 0.14) and there was no correlation between behavior during testing and total Akt expression in this PFC subregion [*r* = −0.131, *p* = 0.301]. However, akin to our prior results for both the 6-h (16) and mixed-access model (47), we observed group differences in a time-dependent increase in p(Ser473)-Akt1 expression within the PL as indexed either by total phospho-protein expression (Figure 4B) [Group X Withdrawal: *F*_(2, 64)_ = 8.12, *p* = 0.001] or the relative expression of the phospho-protein (Figure 4C) [Group X Withdrawal: *F*_(2, 63)_ = 5.354, *p* = 0.007]. Indeed, deconstruction of these interactions along the Group factor indicated that both cocaine IV-SA groups exhibited a time-dependent rise in p(Ser473)-Akt1 levels that was not apparent in Sham controls (Figure 4B) [tests for simple effects: for Mixed: *p* < 0.05; for 2-H, *p* < 0.05; for Sham: *p* > 0.05], while only 2-H rats exhibited a time-dependent increase in relative phospho-protein expression (Figure 4C) [tests for simple main effects: for Mixed, *p* > 0.05; for 2-H: *p* < 0.05; for Sham, *p* > 0.05]. Moreover, akin to our prior findings from the mixed model (47), a trend for a positive correlation between PL p(Ser473)-Akt1 expression and cue-elicited responded was noted [*r* = 0.229, *p* = 0.068], although no correlation was detected between responding and the ratio of phosphorylated vs. total Akt1 expression [*r* = 0.150, *p* = 0.237]. Such findings further implicate increased Akt1 phosphorylation/activation within the PL in the expression of incubated cocaine craving, corroborating prior results from our group (33, 47).

Examination of changes in CaMKII expression within the PL detected no group or time-related differences for total CaMKII expression within the PL (Figure 4D; Group X Withdrawal ANOVA, all *p*'s > 0.616). However, a significant Group X Withdrawal interaction was noted for both the total levels of p(Thr286)-CaMKII (Figure 4E) [*F*_(2, 59)_ = 6.243, *p* = 0.004] and its relative expression within the PL (Figure 4F) [*F*_(2, 59)_ = 5.355, *p* = 0.008]. For both measures, the interactions reflected a time-dependent increase only in the Mixed model [tests for simple main effects for p(Thr286)-CaMKII: for Sham, *p* > 0.05; for 2-h, *p* > 0.05; for Mixed, p < 0.05; tests for simple main effects for the phospho:total ratio: for Sham, *p* > 0.05; for 2-h, *p* > 0.05; for Mixed, *p* < 0.05]. In contrast to p(Ser473)-Akt1, no significant correlations were detected between cue-elicited responding and the levels of total CaMKII (*r* = 0.088, *p* = 0.492), p(Thr286)-CaMKII (*r* = 0.189, *p* = 0.148) or their relative expression (*r* = 0.058, *p* = 0.658) within the PL. These data provide the first indication that CaMKII activation within the PL may be involved in incubated cocaine seeking, although the distinction in the temporal patterning of p(Thr286)-CaMKII (Figures 4E, F) vs. glutamate receptor expression within this subregion (Figures 2, 3) indicates that the activational state of CaMKII within the PL is dissociable from changes in either mGlu or iGlu receptor expression.

### Immunoblotting in the infralimbic cortex (IL)

#### Glutamate receptors and Homer proteins

**Figure 5:**
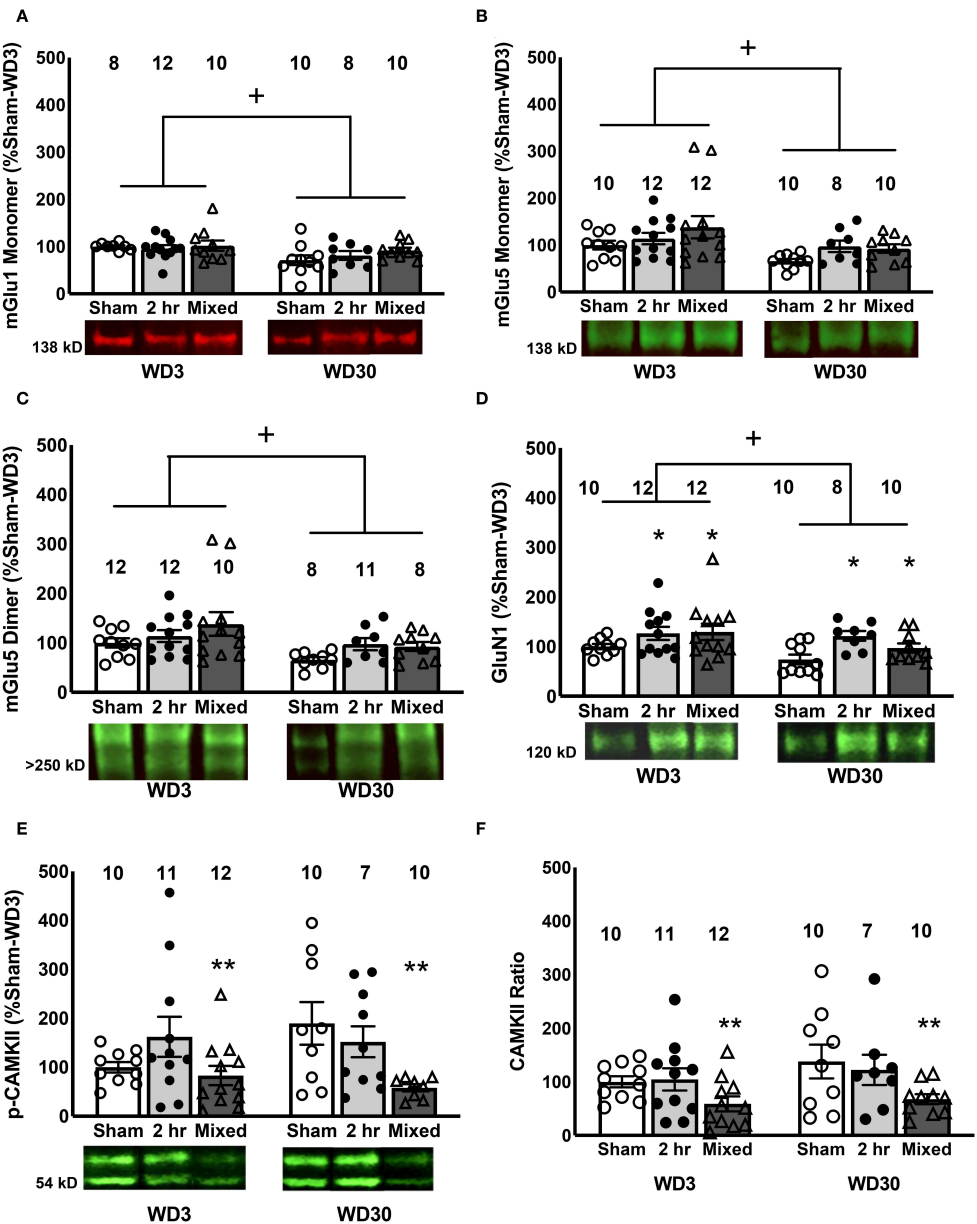
Changes in glutamate receptors and Homer proteins in the IL. Within the IL, we detected a time-dependent reduction in the mGlu1 monomer **(A)**, mGlu5 monomer **(B)**, mGlu5 dimer **(C)** and GluN1 **(D)**, irrespective of the cocaine history of the rats, although both IV-SA groups exhibited higher GluN1 expression than Sham controls. Overall, mixed rats exhibited lower levels of both total **(E)** and **(F)** relative p(Thr286)-CaMKII expression within the IL, relative to both 2-h rats and Sham controls. Data are normalized to the average protein densities of the Sham controls tested on WD3 and represented as means ± SEMs of the number of rats indicated. *p < 0.05, compared to Sham; +*p* < 0.05, compared to WD3; **p < 0.05, compared to other two groups.

In the IL, we detected a time-dependent reduction in the levels of the monomer forms of both Group 1 mGlu receptors, as well as the dimer form of mGlu5, irrespective of the cocaine history of the rats (Figures 5A–C) [for mGlu1, Withdrawal effect: *F*_(1, 57)_ = 7.369, *p* = 0.009; Group effect and interaction, *p*'s > 0.633; for mGlu5 monomer, Withdrawal effect: *F*_(1, 61)_ = 7.203, *p* = 0.01; Group effect and interaction, *p*'s > 0.078; for mGlu5 dimer, Withdrawal effect: *F*_(1, 60)_ = 6.995, *p* = 0.011; Group effect and interaction, *p*'s > 0.165]. However, consistent with Chiu et al. (47), no correlations were detected between cue-elicited responding on test day and the IL expression of either mGlu receptor monomer [for mGlu1, *r* = 0.058, *p* = 0.664; for mGlu5, *r* = −0.147, *p* = 0.253] or the mGlu5 dimer (*r* = −0.161, *p* = 0.215). GluN1 expression was also lower overall at the 30-day withdrawal time-point [Withdrawal effect: *F*_(1, 61)_ = 4.596, *p* = 0.036], but was higher overall in both cocaine IV-SA groups vs. Sham controls (Figure 5D) [Group effect: *F*_(1, 61)_ = 4.908, *p* = 0.011; interaction: *p* = 0.506; SNK tests: 2-h = Mixed>Sham]. No correlation was detected between IL GluN1 expression and behavior [*r* = 0.087, *p* = 0.500].

In contrast, we detected no significant cocaine- or withdrawal-related changes in the expression of any of the other glutamate-related proteins examined herein (see Table 1) [Group X Withdrawal ANOVAs: for Homer1b/c, all *p*'s > 0.120; for Homer2a/b, all *p*'s > 0.150; for GluN2a, all *p*'s > 0.450; for GluN2b, all *p*'s > 0.510, for GluA1, all *p*'s > 0.400; for GluA2, all *p*'s > 0.500) nor did we detect any significant correlations between our glutamate-related proteins and cue-elicited responding on test day (for Homer1b/c, *r* = −0.223 *p* = 0.077; for Homer2a/b, *r* = −0.122, *p* = 0.339; for GluA1, *r* = −0.150, *p* = 0.257; for GluA2, *p* = 0.009, *p* = 0.947; for GluN2a, *r* = −0.004, *p* = 0.973; for GluN2b, *r* = −0.002, *p* = 0.987]. Thus, consistent with our findings from the mixed self-administration model (47), the incubation of cocaine craving is dissociated from changes in the expression of Group1 mGlu receptor-related proteins within the IL, with additional evidence for a dissociation also from changes in the expression of iGlu receptors within this PFC subregion.

**Table 1:** Summary of the negative results from the immunoblotting study of the IL from Sham, 2-h- and Mixed-Access rats.

**Protein**	**WD3**			**WD30**		
	**Sham**	**2-h**	**Mixed**	**Sham**	**2-hr**	**Mixed**
mGlu5	100.00 ± 9.27 (10)	113.91 ± 12.06 (12)	138.13 ± 23.94 (12)	65.95 ± 4.84 (10)	97.55 ± 12.24 (8)	92.77 ± 8.94 (10)
Homer1b/c	100.00 ± 7.87 (10)	73.54 ± 7.18 (12)	88.95 ± 8.70 (12)	95.50 ± 14.63 (10)	95.50 ± 9.74 (9)	72.76 ± 14.63 (10)
Homer2a/b	100.00 ± 5.28 (10)	89.65 ± 11.55 (12)	75.02 ± 7.99 (12)	82.69 ± 8.40 (10)	75.50 ± 12.51 (9)	94.73 ± 14.40 (11)
GluA1	100.00 ± 15.10 (10)	136.17 ± 16.87 (12)	147.81 ± 24.16 (12)	121.27 ± 49.81 (10)	136.60 ± 22.67 (8)	144.61 ± 21.45 (10)
GluA2	100.00 ± 4.42 (10)	116.87 ± 11.31 (12)	139.43 ± 17.46 (12)	142.40 ± 37.86 (10)	115.08 ± 14.19 (8)	130.90 ± 20.71 (10)
GluN2a	100.00 ± 12.40 (10)	94.31 ± 12.05 (12)	125.29 ± 16.56 (12)	165.52 ± 74.21 (10)	88.29 ± 7.87 (8)	128.67 ± 29.91 (10)
GluN2b	100.00 ± 8.37 (10)	89.66 ± 8.23 (12)	121.05 ± 20.49 (11)	129.84 ± 41.79 (10)	95.75 ± 14.24 (8)	102.16 ± 13.73 (10)
Akt1	100.00 ± 4.91 (10)	87.40 ± 11.91 (12)	87.74 ± 11.12 (12)	85.95 ± 7.42 (10)	83.76 ± 22.03 (9)	81.54 ± 9.09 (11)
p(Ser273)-Akt1	100.00 ± 3.53 (10)	83.22 ± 7.70 (12)	94.48 ± 7.27 (12)	99.09 ± 8.15 (10)	91.17 ± 16.34 (9)	110.75 ± 10.41 (11)
p(Ser273)-Akt1:Akt1	100.00 ± 6.59 (10)	104.20 ± 6.59 (12)	125.86 ± 17.57 (12)	118.13 ± 10.46 (10)	133.46 ± 29.39 (9)	147.45 ± 15.64 (11)
CaMKII	100.00 ± 5.87 (10)	141.84 ± 17.19 (12)	194.44 ± 66.93 (12)	128.89 ± 18.15 (10)	153.47 ± 29.91 (8)	104.17 ± 9.98 (10)

The data are presented as the means ± SEMs of the number of rats indicated in parentheses.

#### Akt1 and CaMKII

We also failed to detect significant group differences in the levels of phosphorylated or total Akt1 nor did we detect a difference in their relative expression within the IL (Table 1) [for p(Ser473)-Akt1, all *p*'s > 0.22; for total Akt1, all *p*'s > 0.41; for ratio, all *p*'s > 0.073]. Interestingly, while cue-elicited responding was not correlated with the expression of either total or phosphorylated Akt1 [for total Akt1, *r* = −0.78, *p* = 0.538; for p(Ser473)-Akt1, *r* = −0.126, *p* = 0.326], their relative expression was positively correlated with cue-elicited responding [*r* = 0.314, *p* = 0.012]. This latter result is consistent with our results from Chiu et al. (47), indicating that Akt1 activity within the IL is a molecular correlate of cue-elicited cocaine seeking behavior.

Although we detected no changes in total CaMKII expression within the IL (Table 1) [Group X Withdrawal ANOVA, all *p*'s > 0.167]. A Group effect was detected for p(Thr286)-CaMKII expression within the IL as indexed by either total (Figure 5E) [*F*_(1, 59)_ = 5.017, *p* = 0.010] or relative phospho-protein expression (Figure 5F) [*F*_(1, 59)_ = 5.842, *p* = 0.005]. For both measures, the Group effect reflected lower phospho-protein expression within the IL of Mixed rats, relative to both Sham and 2-H rats (SNK *post-hoc* tests). However, we detected no time-related change in p(Thr286)-CaMKII expression to align with changes in mGlu1 or GluN1 [for p(Thr286)-CaMKII, Withdrawal effect and interaction, *p*'s > 0.132; for phospho:total ratio, *p*'s > 0.180] and no correlations were observed between cue-elicited drug-seeking behavior on test day and the IL expression of CaMKII (*r* = 0.017, *p* = 0.894), p(Thr286)-CaMKII (*r* = −0.011, *p* = 0.936) or their relative expression (*r* = 0.055, *p* = 0.676). As the reduction in CaMKII activity within the IL of Mixed rats was time-independent, it is not likely that reduced IL CaMKII activity contributes to the expression of incubated craving.

## Discussion

Herein, we determined whether or not the PFC protein profile of rats expressing incubated cocaine craving following more procedurally facile and less time-intensive IV-SA procedures recapitulated that observed in our prior immunoblotting studies of incubated cocaine craving under the more typical 6-h or extended-access IV-SA procedure [e.g., (13)]. We also extended prior results to changes in iGluR protein expression and CaMKII phosphorylation, the latter to index calcium-dependent signaling. As summarized in Table 2, we identified increased p(Ser473)-Akt1 and Homer2a/b expression within the PL as common across our immunoblotting studies of incubated cocaine craving to date and provide new evidence that incubated cocaine craving is associated also with increased p(Thr286)-CaMKII levels within the PL. Below, we discuss the present behavioral and immunoblotting findings within the context of the extant literature on incubated cocaine craving and their implications for future research.

**Table 2:** A comparison of protein expression within the PL and IL subregions of the PFC determined in cocaine-experienced rats following our 2-h or mixed-access IV-SA procedures vs. those reported previously in our recent study using the mixed model [Mixed 2021; (47)] and those reported for the entire vmPFC of cocaine-incubated rats following a 6-h IV-SA procedure [6-H (vmPFC)].

	**Prelimbic cortex**				**Infralimbic cortex**		
	**2-H**	**Mixed**	**Mixed (2021)**	**6-H (ventral PL + IL)**	**2-H**	**Mixed**	**Mixed (2021)**
Incubation	Yes	Yes	Yes	Yes	Yes	Yes	Yes
mGlu1 monomer	–	–	↓ (n.s.)	↓^a^	↓ (WD)		↑
mGlu5 monomer	–	–	↓ (n.s.)	↓^a^	↓ (WD)		–
mGlu5 dimer	↓ (WD)		↓ (n.s.)	n.d.	↓ (WD)		–
Homer1b/c	–	–	↑	–^b^	–	–	–
Homer2a/b	↑ (n.s.)	↑	↑	↑^b^	–	–	–
t-Akt	–	–	–	–^c^	–	–	–
p-Akt	↑	↑	↑ (n.s.)	↑^c^	–	–	–
p:t-Akt ratio	↑	–	n.d.	↑^c^	–	–	n.d.
GluA1	–	–	n.d.	n.d.	–	–	n.d.
GluA2	–	–	n.d.	n.d.	–	–	n.d.
GluN1	–	–	n.d.	n.d.	↓ (WD) ↑ (IV-SA)		
GluN2a	–	–	n.d.	n.d.	–	–	n.d.
GluN2b	–	–	n.d.	n.d.	–	–	n.d.
t-CaMKII	–	–	n.d.	n.d.	–	–	n.d.
p-CaMKII	–	↑	n.d.	n.d.	–	↓	n.d.
p:t-CaMKII ratio	–	↑	n.d.	n.d.	–	↓ (IV-SA)	n.d.

a Ben-Shahar et al. (27).

b Gould et al. (28).

c Szumlinski et al. (16).

↑ Denotes an incubation-selective increase in protein expression; ↓ denotes an incubation-selective decrease in protein expression; – denotes no incubation-associated change in protein expression. ↓ WD denotes an overall reduction in protein expression from WD3 to WD30, independent of cocaine self-administration history; ↑IV-SA denotes an overall increase in protein expression, independent of withdrawal time-point. As our prior immunoblotting studies did not assay for iGluR subunits or CaMKII expression, n.d. indicates not determined. n.s. denotes a strong, but non-significant, trend in the data (i.e., p = 0.051–0.090).

### Incubated cocaine craving under short-access IV-SA procedures

In the present study, we detected higher levels of cue-elicited cocaine seeking on WD30 vs. WD3 under both 2-h and Mixed IV-SA procedures, indicating that both short-access procedures elicited an incubation of cocaine craving. The behavioral results for the Mixed model replicate our recent study using the same procedure (47) and align with prior studies employing mixed IV-SA procedures commencing with an initial over-night IV-SA session (39, 40, 43). Likewise, our behavioral results for the 2-h procedure align with prior reports in the literature demonstrating that daily 2-h sessions are sufficient to induce incubated cocaine craving (36, 37) and can do so in the absence of any prior lever-response training for food reinforcement [Figure 1H; (36)]. Thus, it would appear that daily 2-h IV-SA sessions are sufficient to instigate whatever neuroadaptations drive the expression of incubated cocaine craving.

This being said, it is worth noting that while both IV-SA group exhibited an incubation of cocaine-craving, Cohen's *d* analyses indicated that the effect size was larger in rats that underwent the Mixed vs. 2-h procedure due to the relatively high level of responding exhibited by the 2-h rats on WD3 (Figure 1H). Interestingly, a prior comparison between the effect of 2- vs. 12-h daily sessions on subsequent incubated cocaine seeking reported higher responding on 1, 10, and 60 days withdrawal in the shorter vs. longer access condition (despite the latter consuming substantially more cocaine) (36). Such findings, coupled with repeated demonstrations in the literature that a time-dependent intensification of cue reactivity does not require repeated sessions of extended (i.e., 6+ h/day) cocaine-access to manifest (35, 37, 39–43, 47), indicate that the amount of prior cocaine consumption is not necessarily predictive of the magnitude of cocaine cue reactivity nor its capacity to incubate during protracted withdrawal, raising the question of whether or not common or unique neuroadaptations drive the incubated state under shorter- vs. longer-access procedures.

### Cue-elicited cocaine craving is not overtly related to changes in total iGlu receptor expression within PFC subregions

A large body of evidence exists indicating that the profile of neural adaptions induced by extended-access IV-SA procedures are distinct from those induced under short-access (i.e., 1 or 2 h/day) procedures [e.g., (48, 55–62)]. However, there is accumulating evidence from studies of the NAC core that the incubation of cocaine craving is linked to specific glutamate perturbations within this region (notably, reduced mGlu1 expression/function and increased expression of calcium-permeable, GluA2-lacking AMPA receptors), independent of the specific IV-SA procedure employed to induce the incubated response [e.g., (17, 19, 21–23, 39, 40, 43, 44, 54, 63)]. Thus, we rationalized that if specific neuroadaptations within the mPFC underpin the manifestation of incubated drug-craving, then there should be overlap in the mPFC protein profile observed under distinct IV-SA models that induce cocaine-incubated responding.

Herein, we failed to detect any incubation-specific changes in iGlu receptor subunit expression within either PFC subregion in rats tested on WD30 (Table 2). This is our first examination for incubation-related changes in iGlu receptor expression within PFC subregions and as such, we employed conventional immunoblotting procedures on whole-cell lysates that cannot inform as to the membrane localization of our receptor subunits or the functional status of the receptors. Thus, our failure to detect incubation-associated changes in iGlu receptor subunit expression within PFC may reflect a lack of subcellular specificity in our results or regional distinctions in the glutamate-related effects of cocaine withdrawal as reported in the literature [e.g., (48, 64–66); see review (67)]. Alternatively, a recent study implicated time-dependent changes in the plasma membrane expression of the magnesium-insensitive and calcium-impermeable GluN3 subunit of the NMDA receptor within the NAC core as important for incubated cocaine craving (44). This NMDA subunit was not examined herein due to limited tissue but presents yet additional protein target for future studies probing the relationship between incubated cocaine craving and the plasma membrane expression of glutamate receptor-related proteins.

### Protein correlates of incubated cocaine craving are selective for the PL

As is apparent from Table 2, several patterns emerge from a comparison of our immunoblotting results to date for the mPFC or its subregions. First, incubated cocaine craving is paralleled by changes in glutamate-related protein expression more selectively within the PL vs. the IL [(47); present study], and the incubation-associated changes in PL protein expression align with those reported for the entire vmPFC (i.e., ventral PL and IL combined) in earlier studies using a 6-h IV-SA model [Table 2; (27, 28, 33)]. The observation that cocaine-incubated rats express a larger number of glutamatergic anomalies in the PL vs. the IL aligns with the relative densities of their projections to the NAC core [e.g., (68)]—a neural locus key to the expression of incubated cocaine [c.f., (15, 69)]. Our immunoblotting findings also align with optogenetic evidence indicating that PL and IL projections to NAC subregions undergo distinct forms of glutamate receptor-dependent synaptic plasticity that drive and inhibit, respectively, the expression of incubated cocaine craving (40). Our immunoblotting findings are also consistent with our prior neuropharmacological studies in which targeting the PL with receptor antagonists, kinase inhibitors or adeno-associated viruses exerted effects in cocaine-experienced rats tested during protracted withdrawal (27–29, 33). It should be noted that, in contrast to our prior studies using the 6-h model [e.g., (27, 28, 33)], our more recent studies employing shorter cocaine-access procedures [e.g., (47); present study] did not include control groups with equivalent cocaine history, but did not undergo testing for cue-elicited drug-seeking. While one might argue that the incubation-related protein changes observed in these more recent studies might merely reflect an effect of cocaine withdrawal, the fact that we failed to detect changes in Group1 mGlu receptor or Homer expression (27, 28), nor did we detect indices of kinase activation (29, 33) within the mPFC of rats at either 3 or 30 days withdrawal from with a 10-day history of 6-h cocaine-access, argues against these protein changes as merely reflecting neuroadaptations to cocaine withdrawal. While we did not discriminate between the PL and IL in our prior immunoblotting studies of the 6-h cocaine-access model, the fact that no changes in protein expression were detected within the mPFC of 6-h rats left undisturbed in the home-cage argues that cue re-exposure, rather than the amount of cocaine self-administered prior to withdrawal, is a major driving factor for incubation-related changes in AKT activation and Homer2 expression. This being said, it would be important in future studies to confirm this is the case by including control rats with equivalent shorter-access cocaine self-administration history that are not tested for cue-elicited drug-seeking behavior.

### PL group 1 mGlu receptor expression and the manifestation of incubated cocaine craving

From a comparison of findings across our studies to date (Table 2), it would appear that the 6-h model induces a more robust and reliable reduction in Group 1 mGlu receptor expression than the shorter-access models. While consistent with reported distinctions in the neurobiological profile of rats following short- vs. extended-access IV-SA procedures [e.g., (48, 55–61), neuropharmacological manipulations of neither mGlu1 nor mGlu5 activity within the vmPFC impact the expression of incubated cocaine craving during an initial test of cue reactivity (27). Thus, reduced mGlu1 or mGlu5 function/expression within the mPFC does not drive cocaine-incubated response *per se*. Instead, our neuropharmacological results indicate that reduced vmPFC mGlu1 and mGlu5 expression impairs the capacity to consolidate extinction learning that occurs during an initial cue reactivity test, thereby promoting or prolonging the incubated state (27). As the rats in the present study were euthanized immediately following the cue test, we did not determine how specific IV-SA procedures impact the consolidation of extinction learning. That being said, incubated cocaine craving persisted across days in our recent study using the mixed-access procedure and these rats exhibited a strong trend toward lower PL expression of both mGlu1 and mGlu5, relative to their WD3 controls [Table 2; (47)]. Given the therapeutic potential of positive allosteric modulators of Group1 mGlu receptors for attenuating incubated cocaine craving [see (21, 23, 63, 70, 71); see also (72)] and its persistence in the face of repeated cue re-exposure and associated synaptic plasticity (27), it will be important to determine in future studies whether mGlu1 and mGlu5 receptors within PFC gate the consolidation of extinction learning under other IV-SA procedures that are sufficient to elicit incubated craving and to study the mechanism(s) through which this gating occurs.

### PL Homer2a/b expression is biochemical correlate of cue-elicited cocaine craving

Another pattern across our incubation studies is a robust increase in the PL/vmPFC expression of the glutamate receptor scaffolding protein Homer2a/b [Table 2; (28, 47)] and its positive correlation with the magnitude of cue-reactivity on test day [(29, 47); present study]. Herein, a more robust increase in PL Homer2a/b expression paralleled the more robust incubated cocaine craving in the Mixed rats, relative to the 2-h rats (Figure 2D). The relative robustness of the Homer2a/b effect in Mixed vs. 2-h rats presumably reflects the higher drug intake exhibited by Mixed rats on Day 1 of self-administration when the rats had 6 h-access to IV cocaine (Figure 1C), as their cocaine self-administration behavior over the last 3 days of IV-SA training were comparable to that of the 2-h group (Figures 1E–G). If this is in fact the case, then a single 6-h cocaine IV-SA session may be sufficient to “jump-start” or augment cocaine-dependent changes in Homer2a/b scaffolding. The question remains as to why changes in Homer2a/b scaffolding are not apparent until protracted withdrawal, irrespective of the duration of cocaine self-administration [(28); present study].

Our results suggest a potential role for PL Homer2a/b in modulating the magnitude or persistence of incubated cocaine craving, possibly *via* its capacity to regulate Group1 mGlu or NMDA receptor synaptic localization and function [c.f., (73, 74)]. However, arguing against this possibility, virus-mediated knock-down of Homer2b expression within the vmPFC (primarily PL) during withdrawal from a 6-h cocaine IV-SA procedure failed to impact the magnitude of incubated responding on WD30 (28). Likewise, Homer2b knock-down within the vmPFC does not alter the expression of a cocaine-conditioned place-preference by mice (75). While these prior findings indicate that vmPFC Homer2b expression is not necessary for the expression of cocaine-conditioned behavior under either operant- or place-conditioning procedures, Homer2b over-expression within vmPFC of mice shifts the dose-response function for a cocaine-conditioned place-preference to the left (75). Thus, elevated vmPFC Homer2b expression is sufficient to augment the positive affective/motivational valence of cocaine-paired contexts. Homer2b over-expression within the vmPFC also elevates basal extracellular glutamate levels within this region (75) and glutamate release within both the PL and IL is required for the expression of incubated cocaine craving (31). Further, Homer2b over-expression within the vmPFC also induces “cocaine-like” anomalies in both pre- and post-synaptic aspects of glutamate signaling within the NAC of drug-naïve mice [incl. reduced basal extracellular glutamate content, Group1 mGlu receptors and Homer proteins, in addition to potentiated cocaine-induced glutamate release; (75)], which could also promote cocaine cue reactivity and its incubation during protracted withdrawal [c.f., (72, 76)]. However, it remains to be determined whether or not vmPFC Homer2b over-expression is sufficient to drive cue reactivity and its incubation under operant-conditioning procedures.

### Phosphorylated Akt1 is a biochemical correlate of cue-elicited cocaine seeking that is reliably associated with the expression of incubated cocaine craving

Converging pharmacological evidence supports a key role for Akt1/PI3K/mTOR signaling in the expression of incubated cocaine craving (25, 33, 47, 77). As summarized in Table 2, increased PL/vmPFC expression of p(Ser473)-Akt1 is another protein change associated with the expression of incubated craving [Figure 4B; (33, 47)] and positively correlated with cue-reactivity on test day [(47); present study]. In our recent study of incubated cocaine craving under the mixed-access model (47), increased p(Ser473)-Akt1 expression within the PL coincided within increased phosphorylation of the mTOR effector p(Thr389)-P70S6 kinase, as well as its target, p(Ser234/235)-riboprotein S6. Unfortunately, repeated attempts to immunoblot for these markers of mTOR activation herein were unsuccessful; thus, it remains to be determined whether or not these particular immunoblotting findings replicate across studies or models. However, it should be noted that a single oral pretreatment with the mTOR inhibitor Everolimus is sufficient to block incubated cocaine craving under our mixed-access procedure and the effect lasted for at least 24 h (47). The enduring Everolimus effect on incubated cocaine craving is reminiscent of the effects of an intra-vmPFC infusion of the PI3K inhibitor wortmannin on incubated responding expressed under 6-h IV-SA procedures, which also persisted for at least 24 h (33), arguing an important role for signaling pathways involving Akt1/PI3K and mTOR within vmPFC in driving the incubated state. Consistent with this, Everolimus pretreatment significantly lowered the PL expression of Homer2a/b, p(Ser473)-Akt1 and p(Thr389)-P70S6 kinase—three biomolecules associated with the cocaine-incubated state [Table 2; (47)].

Inhibition of mTOR signaling impairs protein translation and this mechanism has been implicated in cue-elicited cocaine craving and its incubation (25). Whether or not the capacity of systemic Everolimus or intra-vmPFC wortmannin to block incubated cocaine craving involves an inhibition of protein translation within PFC subregions has not been examined to date. However, it is worth noting that Everolimus significantly *increases* Group 1 mGlu receptor expression within the PL on WD30, coincident with reduced Akt1 phosphorylation, indices of mTOR activation, and Homer2a/b expression, with no significant changes in the expression of the non-phosphorylated forms of any signaling molecules examined or Homer1b/c (47). From these limited data, it would appear that if an inhibition of protein translation is a mechanism through which PI3K and mTOR inhibitors block incubated cocaine craving, there is specificity to this inhibition and it will be important in future work to decipher what specific proteins are affected and what signals direct this specificity.

### Activated CaMKII in the PL is associated with incubated cocaine craving under the mixed model

The calcium-dependent kinase CaMKII has long been suggested as a potential mediator of cocaine reinforcement and reward [c.f., 77, 78, 99] and studies have implicated the activational state of CaMKII (78), as well as its ability to bind and inhibit diacylglycerol lipase-α (79) in incubated cocaine craving. However, in contrast to the earlier immunoblotting study of Caffino et al. (78), in which rats self-administered IV cocaine for 2 h/day for 14 days prior to withdrawal, we failed to detect any increase in CaMKII auto-phosphorylation [indexed by the levels of p(Thr286)-CaMKII] within either the PL or the IL of cocaine-experienced rats tested in early cocaine withdrawal. In fact, PL levels of p(Thr286)-CaMKII tended to be lower in the two cocaine IV-SA groups on WD3, relative to Sham controls (Figure 4F) and Mixed rats exhibited significantly lower p(Thr286)-CaMKII expression within the IL than either of other two experimental conditions at this earlier withdrawal time-point (Figures 5E, F). The discrepancy in results between this and the earlier study of Caffino et al. (78) may reflect the fact that the rats in the present study underwent a cue test session immediately prior to tissue collection, while those in Caffino et al. (78) simply remained in the home cage for their designated withdrawal period. In support of this, many reports indicate that the vmPFC protein profile of cocaine-withdrawn rats varies as a function of re-exposure to drug-associated cues and/or contexts [e.g., (27–29, 33, 66)].

However, we did detect a significant increase in the PL expression of p(Thr286)-CaMKII on WD30, but this was apparent only in the Mixed rats (Figure 4F). In contrast, these same rats expressed lower p(Thr286)-CaMKII expression within the IL in late withdrawal (Figures 5E, F). Thus, at least under mixed-access procedures, the expression of incubated cocaine craving is associated with opposite changes in the activational state of CaMKII within the PL vs. IL, which is intriguing as PL-NAC core and IL-NAC shell projections purportedly gate drug-seeking behavior in opposite directions [c.f., (80–83)] and presumably, these effects were instigated by the longer initial IV-SA session experienced by the Mixed group as they were not present in the 2-h rats. Admittedly, the observation that our 2-h rats failed to exhibit any time-dependent change in p(Thr286)-CaMKII expression weakens the hypothesis that changes in CaMKII activation within the vmPFC are required for the manifestation of incubated cocaine craving, but does not necessarily preclude a role for this signaling molecule in CaMKII in gating the magnitude of this phenomenon. Thus, a goal for future work is to determine the functional relevance of these subregion-selective changes in activated CaMKII for both cue-elicited cocaine seeking and its incubation during protracted drug abstinence.

Curiously, no obvious relationships were detected between p(Thr286)-CaMKII expression within either PFC subregion and the expression of glutamate receptor proteins that gate calcium-dependent signaling [e.g., Group1 mGlu receptors, NMDA receptors and GluA2-lacking AMPA receptors; for recent reviews, see: (74, 84, 85)]. Thus, pending the results of behavioral studies of CaMKII function in incubated craving, another important aspect of future work will be to determine which receptors (glutamate or otherwise) might be responsible for the subregion-specific changes in p(Thr286)-CaMKII expression. Related to this, CaMKII drives both the homologous and heterologous desensitization of mGlu1α (86, 87). Of potential relevance to the cocaine-incubated state, a time-dependent increase in activated CaMKII within the PL may contribute, at least in part, to the reduced mGlu1 expression observed in prior studies (27). However, our failure to detect significant reductions in the PL expression of either mGlu1 or mGlu5 under shorter-access IV-SA procedures [(Figure 2); (47)] weakens this possibility. Further, although evidence also supports a physical interaction between CaMKII and mGlu5 in brain (88, 89), p(Thr286)-CaMKII is reported to increase, not reduce, the plasma membrane expression of mGlu5 and calcium-dependent signaling through this receptor (89). Likewise, activated CaMKII is also a known regulator of AMPA [e.g., (90–92)], as well as NMDA [e.g., (93–97)] receptor trafficking, signaling and receptor-dependent synaptic plasticity, furthering the importance of relating the cocaine-incubated state to the plasma membrane expression of iGlu receptors within PFC and determining their relevance for this phenomenon.

### Conclusion

An incubation of cocaine craving can be reliably observed under simple shorter-access IV-SA procedures. Such procedures result in a profile of protein expression within the PL subregion of the PFC that is partially consistent with that reported for longer cocaine IV-SA models, further demonstrating that increased Homer2a/b and p(Ser473)-Akt1 expression are biochemical correlates of cue-elicited cocaine seeking that are associated with the incubated state. The present data also provide evidence that the manifestation of incubated cocaine craving can be dissociated from changes in the total protein expression of Group 1 mGlu, AMPA and NMDA receptors within the PFC, but implicate subregion-selective changes in CaMKII auto-phosphorylation as a potential mediator of incubated cocaine craving.

## Data availability statement

The raw data supporting the conclusions of this article will be made available by the authors, without undue reservation.

## Ethics statement

The animal study was reviewed and approved by Institutional Care and Use Committee of the University of California, Santa Barbara.

## Author contributions

Conceptualization: LH, MS, AC, TK, and KS. Formal analysis and writing—original draft preparation: KS, LH, and MS. Investigation: LH, MS, AC, ES, and GS. Writing—review and editing: LH, MS, AC, ES, GS, TK, and KS. Visualization and project administration: LH and KS. Supervision and funding acquisition: TK and KS. All authors have read and agreed to the published version of the manuscript.

## Supplementary material

Supplementary Figure 1 Representative immunoblots for mGlu1. The commercial anti-mGlu1 antibody employed in this study detected only a single band at ~138 kD in both PL and IL tissue.
