# Supplementary figures and images for: Profiling prefrontal cortex protein expression in rats exhibiting an incubation of cocaine craving following short-access self-administration procedures
Source: Front Psychiatry. 2023 Jan 4;13:1031585. doi: 10.3389/fpsyt.2022.1031585 (PMC9846226; doi:10.3389/fpsyt.2022.1031585)

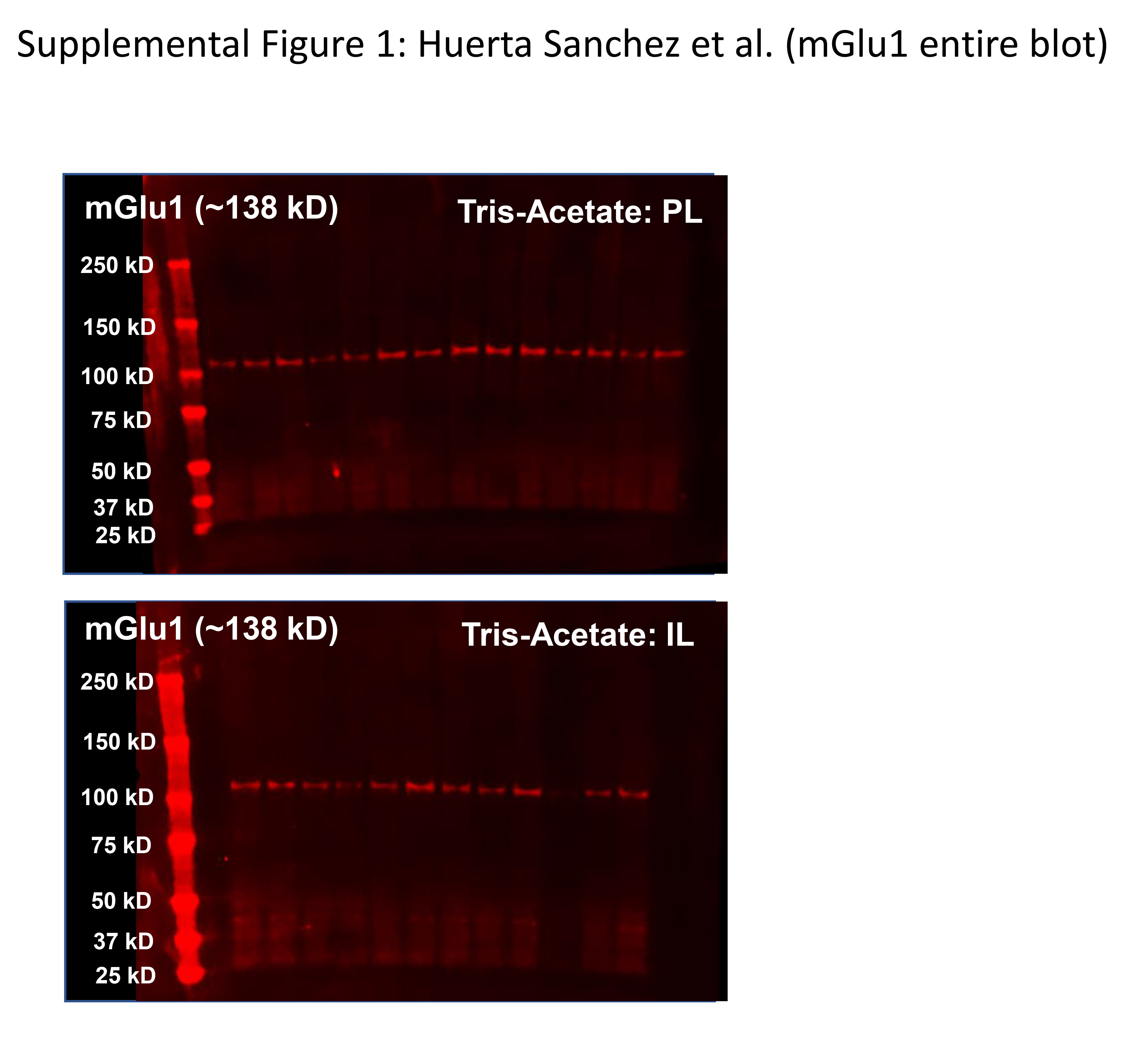

Supplement: Supplementary Figure 1 — Representative immunoblots for mGlu1. The commercial anti-mGlu1 antibody employed in this study detected only a single band at ~138 kD in both PL and IL tissue. [file Image_1.JPEG]
